# Supplementary figures and images for: Mosquito surveillance on U.S military installations as part of a Japanese encephalitis virus detection program: 2016 to 2021
Source: PLoS Negl Trop Dis. 2023 Oct 19;17(10):e0011422. doi: 10.1371/journal.pntd.0011422 (PMC10617694; doi:10.1371/journal.pntd.0011422)

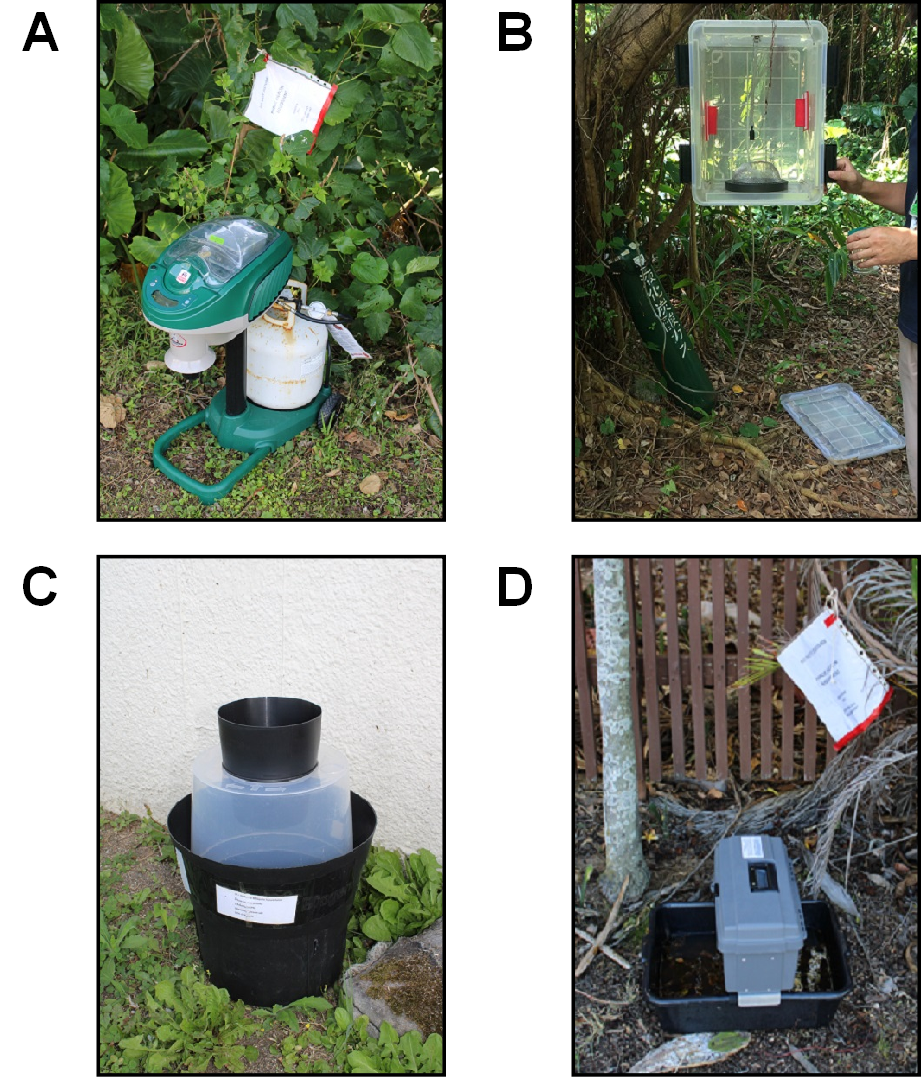

Supplement: S1 Fig — A–Mosquito Magnet; B–Passive Box Trap; C–Biogents Gravid Autocidal Trap; D–Reiter/Cummings Gravid Trap. All photos were taken by the author, Dr. Mark F. Olson. (TIF) [file pntd.0011422.s001.tif]

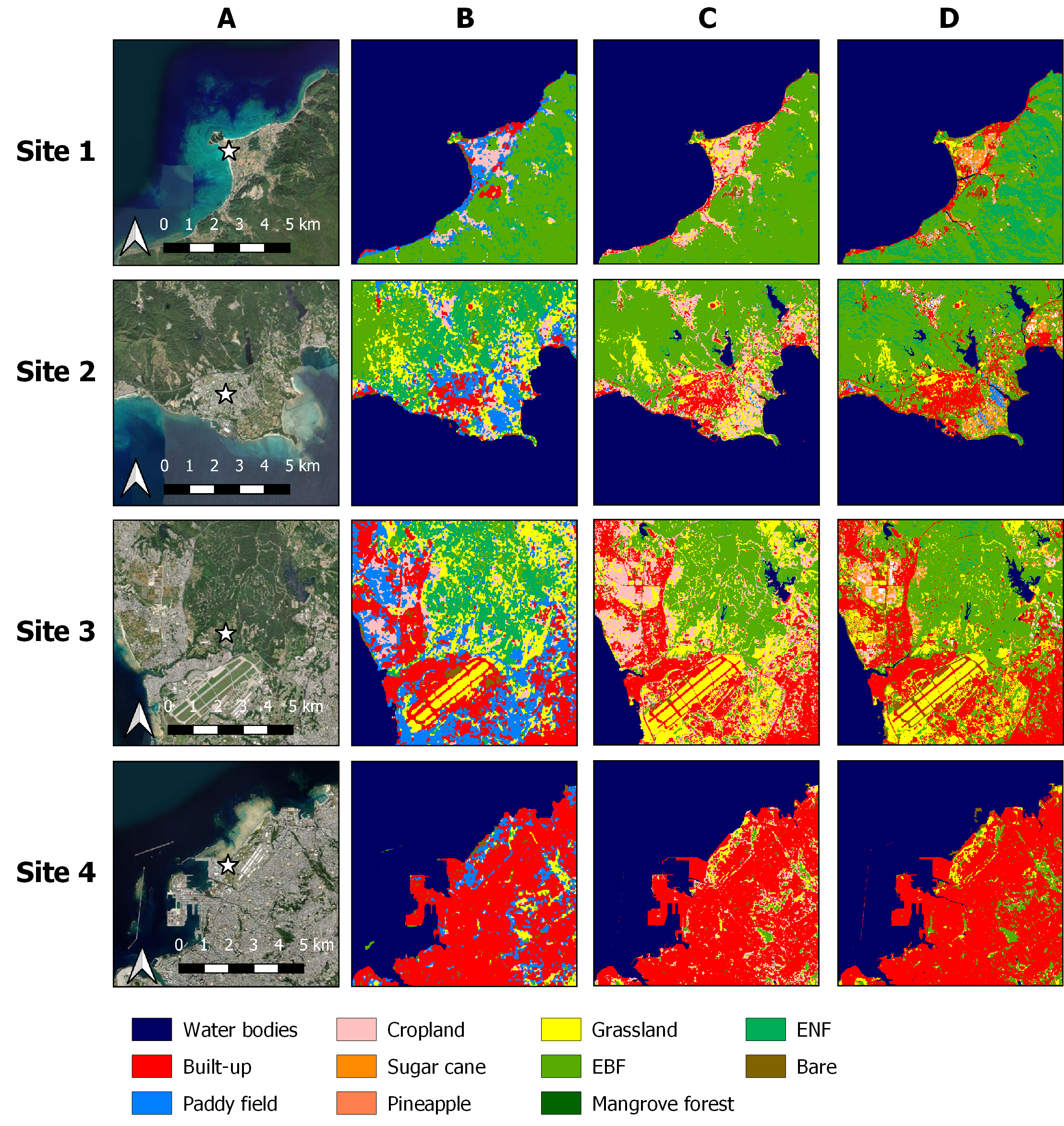

Supplement: S2 Fig — A = Satellite imagery showing trap locations. Content is the intellectual property of Esri and is used herein with permission. Copyright 2023 Esri and its licensors. All rights reserved. B = Land use around 2015. C = Land use around 2019. D = Land use around 2020 (latest version). (Source: ALOS–Advanced Land Observing Satellite, Research and Application Project; https://www.eorc.jaxa.jp/ALOS/en/dataset/lulc_e.htm; Content is the intellectual property of Japan Aerospace Exploration Agency (JAXA) and is used herein with permission). (TIF) [file pntd.0011422.s002.tif]
