# Supplementary material for: Mosquito surveillance on U.S military installations as part of a Japanese encephalitis virus detection program: 2016 to 2021
Source: PLoS Negl Trop Dis. 2023 Oct 19;17(10):e0011422. doi: 10.1371/journal.pntd.0011422 (PMC10617694; doi:10.1371/journal.pntd.0011422)
Supplement: Attachment — Submitted filename: Response to reviewers.docx [file pntd.0011422.s004.docx]

Reviewer's Responses to Questions

Key Review Criteria Required for Acceptance?
As you describe the new analyses required for acceptance, please consider the following:

**Methods**
-Are the objectives of the study clearly articulated with a clear testable hypothesis stated?
-Is the study design appropriate to address the stated objectives?
-Is the population clearly described and appropriate for the hypothesis being tested?
-Is the sample size sufficient to ensure adequate power to address the hypothesis being tested?
-Were correct statistical analysis used to support conclusions?
-Are there concerns about ethical or regulatory requirements being met?

Reviewer #1: Methods are appropriate to test the hypothesis, although more information in some sections would be beneficial to strengthen the data.

1. Line 95: Are there any mosquito species bias using the passive box traps? Please include the answer/explanation in the text.

Author response: Thank you for this question. Trap bias toward various mosquito species has been documented and comparative trials prior to future research to ensure target vectors are effectively captured is recommended. (Please see additional explanation added in lines 287-289.

2. Line 101-102: Delete "The sugar-baited nucleic acid preservation cards."

Author response: Thank you for catching this, it has been deleted.
3. Line 114: Please include how the homogenized mosquito samples were shipped. Temperature? Duration of travel? At certain temperatures, short storage periods in buffer AVL could have a significant effect on the viral RNA integrity.

Author response: The following was added at line 114 and following: “Mosquito lysate samples were shipped to Institute of Molecular Biosciences, Mahidol University, Thailand via FedEx® for molecular analysis and pathogen detection. Samples were shipped at room temperature with an average transit time of 4 days.”

Additionally, the following explanation was added to the Discussion section, starting at line 288.

“Also, homogenized mosquito samples were held at room temperature (24° C) in Buffer AVL for up to 7 days for packaging and shipping to Mahidol University. Recognizing the potential for RNA degradation, our team developed a quality assurance process using positive and negative proficiency analyte testing rounds (blinded to lab personnel) to include with field samples. To date, positive controls undergoing the same packaging and shipping protocol indicate sample integrity is maintained.”

4. Line 134: Some JEV PCR primers are more sensitive to certain genotypes. Any data that this PCR based on genotype III works well to identify others, including the now dominant genotype Ib? Please include this information in the manuscript.

Author response: Agreed. We have added the following (lines 139 to 142) to the revised manuscript “We note that the JEV primers used in this study were based on JEV genotype III sequences, and over time this genotype has largely been replaced by genotype I in Asia (Pan et al., 2011), and as such it is possible that the primers used in the study are less sensitive to detect circulating JEV, although at this point there is no evidence to refute or support this supposition.”

Pan X.L., Liu H., Wang H.Y., Fu S.H., Liu H.Z., Zhang H.L., Li M.H., Gao X.Y., Wang J.L., Sun X.H., et al. Emergence of genotype I of Japanese encephalitis virus as the dominant genotype in Asia. J. Virol. 2011;85:9847–9853. doi: 10.1128/JVI.00825-11.

--------------------

**Results**
-Does the analysis presented match the analysis plan?
-Are the results clearly and completely presented?
-Are the figures (Tables, Images) of sufficient quality for clarity?

Reviewer #1: The results are very clear and concise. The supplementary figures are especially very visually pleasing and able to highlight the important information right away.

1. Figure 1: It would be great if sites 1-4 were labeled on the map.

Author response: Thank you for this recommendation! Sites on Fig 1 have now been labeled and a legend is added below the figure.

2. Line 175: No positive detection by the pan-flavivirus PCR either? Please include this information.

Author response: Agreed. This has been further clarified in the manuscript (lines 178 to 182) “None of the samples screened for JEV specifically showed any amplification products after screening with pan-flavivirus primers. However, while a number of flaviviruses including members of the JEV, Kokobera and Nataya virus serocomplexes of the genus Flavivirus have the same transmission vectors as JEV (Smith, 2017) to the best of our knowledge none of these viruses have ever been reported in Japan.”

Duncan R. Smith (2017) Waiting in the wings: The potential of mosquito transmitted flaviviruses to emerge, Critical Reviews in Microbiology, 43:4, 405-422, DOI: 10.1080/1040841X.2016.1230974

3. Line 193: Please include the appropriate p-values for the Site 3 statement.

Author response: Thank you, p-values for Site 3 have been added.

4. Supplementary figures: Any way to include SFigs 2 and 3 in the main manuscript? They are very helpful as a results summary. Maybe even replace Table 3 or 4 and turn either or both tables as supplementary tables.

Author response: We have added SFigs 2 and 3 to the main manuscript but have decided to keep the tables as well. Thank you for this recommendation.
--------------------

**Conclusions**
-Are the conclusions supported by the data presented?</br>
-Are the limitations of analysis clearly described?</br>
-Do the authors discuss how these data can be helpful to advance our understanding of the topic under study?</br>
-Is public health relevance addressed?</br></br>

Reviewer #1: Please explain on the study limitations (i.e. any mosquito species bias with the selected mosquito traps).

Author response: Study limitations are included in the Discussion section, starting at Line 294.
--------------------

**Editorial and Data Presentation Modifications?**
Use  this section for editorial suggestions as well as relatively minor modifications of existing data that would enhance clarity. If the only modifications needed are minor and/or editorial, you may wish to recommend “Minor Revision” or “Accept”.

Reviewer #1: (No Response)

--------------------

**Summary and General Comments**
Use  this section to provide overall comments, discuss strengths/weaknesses of the study, novelty, significance, general execution and scholarship. You may also include additional comments for the author, including concerns about dual publication, research ethics, or publication ethics. If requesting major revision, please articulate the new experiments that are needed.

Reviewer #1: The authors submitted a well-written manuscript describing their JEV mosquito surveillance in Okinawa, Japan. Adequate background information was provided in both the introduction and conclusion to highlight the importance of the study and to help readers digest the data collected. It would be great if the requested additional information is included in the manuscript to strengthen their results and the readers' understanding.

Author response: Thank you for your review and constructive criticism. Your suggested edits have been incorporated into this final version of the manuscript.

--------------------

PLOS authors have the option to publish the peer review history of their article ("<https://journals.plos.org/plosntds/s/editorial-and-peer-review-process#loc-peer-review-history>" what does this mean?). If published, this will include your full peer review and any attached files.

If you choose “no”, your identity will remain anonymous but your review may still be made public.

Do you want your identity to be public for this peer review? For information about this choice, including consent withdrawal, please see our "<https://www.plos.org/privacy-policy>" Privacy Policy

Reviewer #1: No


Figure Files:
While revising your submission, please upload your figure files to the Preflight Analysis and Conversion Engine (PACE) digital diagnostic tool, [https://pacev2.apexcovantage.com](https://pacev2.apexcovantage.com/). PACE helps ensure that figures meet PLOS requirements. To use PACE, you must first register as a user. Then, login and navigate to the UPLOAD tab, where you will find detailed instructions on how to use the tool. If you encounter any issues or have any questions when using PACE, please email us at [figures@plos.org](mailto:figures@plos.org).

Data Requirements:
Please note that, as a condition of publication, PLOS' data policy requires that you make available all data used to draw the conclusions outlined in your manuscript. Data must be deposited in an appropriate repository, included within the body of the manuscript, or uploaded as supporting information. This includes all numerical values that were used to generate graphs, histograms etc.. For an example see here: <http://www.plosbiology.org/article/info%3Adoi%2F10.1371%2Fjournal.pbio.1001908#s5>.

Reproducibility:
To enhance the reproducibility of your results, we recommend that you deposit your laboratory protocols in [protocols.io](http://protocols.io/), where a protocol can be assigned its own identifier (DOI) such that it can be cited independently in the future. Additionally, PLOS ONE offers an option to publish peer-reviewed clinical study protocols. Read more information on sharing protocols at <https://plos.org/protocols?utm_medium=editorial-email&utm_source=authorletters&utm_campaign=protocols>

References
Please review your reference list to ensure that it is complete and correct. If you have cited papers that have been retracted, please include the rationale for doing so in the manuscript text, or remove these references and replace them with relevant current references. Any changes to the reference list should be mentioned in the rebuttal letter that accompanies your revised manuscript. If you need to cite a retracted article, indicate the article's retracted status in the References list and also include a citation and full reference for the retraction notice.

__________________________________________________
In compliance with data protection regulations, you may request that we remove your personal registration details at any time.  (Use the following URL: <https://www.editorialmanager.com/pntd/login.asp?a=r>). Please contact the publication office if you have any questions.
